# A Phylogeographic Investigation of African Monkeypox

**DOI:** 10.3390/v7042168

**Published:** 2015-04-22

**Authors:** Yoshinori Nakazawa, Matthew R. Mauldin, Ginny L. Emerson, Mary G. Reynolds, R. Ryan Lash, Jinxin Gao, Hui Zhao, Yu Li, Jean-Jacques Muyembe, Placide Mbala Kingebeni, Okito Wemakoy, Jean Malekani, Kevin L. Karem, Inger K. Damon, Darin S. Carroll

**Affiliations:** 1 Poxvirus and Rabies Branch, Centers for Disease Control and Prevention, 1600 Clifton Rd NE, Atlanta, GA 30333, USA; E-Mails: mmauldin@cdc.gov (M.R.M.); gemerson@cdc.gov (G.L.E.); mreynolds3@cdc.gov (M.G.R.); rlash@cdc.gov (R.R.L.); jgao2@cdc.gov (J.G.); hzhao1@cdc.gov (H.Z.); yuli@cdc.gov (Y.L.); kkarem@cdc.gov (K.L.K.); idamon@cdc.gov (I.K.D.); dcarroll@cdc.gov (D.S.C.); 2 Oak Ridge Institute for Science and Education (ORISE) CDC Fellowship Program, Oak Ridge, TN 37831, USA; 3 INRB Laboratory, Avenue de la Démocratie. Kinshasa-Gombe B.P. 1197 Kinshasa 1, Democratic Republic of the Congo; E-Mails: muyembejj@gmail.com (J.-J.M.); mbalaplacide@gmail.com (P.M.K.); 4 Kinshasa School of Public Health, University of Kinshasa, 11850 Kinshasa, Democratic Republic of the Congo; E-Mail: okitow@yahoo.fr; 5 Biology Department, University of Kinshasa, P.O. Box 218 Kinshasa XI, Democratic Republic of the Congo; E-Mail: jean.malekani@unikin.ac.cd

**Keywords:** monkeypox, orthopoxvirus, Phylogenetics, Pleistocene, ecological niche model, evolution

## Abstract

Monkeypox is a zoonotic disease caused by a virus member of the genus *Orthopoxvirus* and is endemic to Central and Western African countries. Previous work has identified two geographically disjuct clades of monkeypox virus based on the analysis of a few genomes coupled with epidemiological and clinical analyses; however, environmental and geographic causes of this differentiation have not been explored. Here, we expand previous phylogenetic studies by analyzing a larger set of monkeypox virus genomes originating throughout Sub-Saharan Africa to identify possible biogeographic barriers associated with genetic differentiation; and projected ecological niche models onto environmental conditions at three periods in the past to explore the potential role of climate oscillations in the evolution of the two primary clades. Analyses supported the separation of the Congo Basin and West Africa clades; the Congo Basin clade shows much shorter branches, which likely indicate a more recent diversification of isolates within this clade. The area between the Sanaga and Cross Rivers divides the two clades and the Dahomey Gap seems to have also served as a barrier within the West African clade. Contraction of areas with suitable environments for monkeypox virus during the Last Glacial Maximum, suggests that the Congo Basin clade of monkeypox virus experienced a severe bottleneck and has since expanded its geographic range.

## 1. Introduction

Monkeypox (MPX) is a zoonotic disease caused by a member of the *Orthopoxvirus* genus, which includes other viruses pathogenic to humans (e.g., variola virus, vaccinia virus, and cowpox virus), and produces mild to severe rash illness in infected individuals. The first human case of monkeypox was identified in the Democratic Republic of the Congo (DRC) in 1971 [1] and is currently a major public health concern in that country. Surveillance of human cases of MPX in Central Africa was very active between 1970 and 1986, particularly at the end of the smallpox eradication campaign, with more than 400 human cases reported during this period [2,3,4]. Since then, MPX surveillance has been limited to investigations of outbreaks of the disease [5,6,7,8,9]. In recent years, MPX surveillance in DRC has increased given the growing interest in clarifying the poorly understood natural history of the virus [10,11,12].

Likos *et al.* [13], based on phylogenetic analyses of monkeypox virus (MPXV) isolates, supported the recognition of two distinct clades of this virus: Western African (WA) and Congo Basin (CB) clades. These two clades are geographically disjunct and have defined epidemiological and clinical differences [7,14,15]. In Likos *et al.* [13], isolates grouped within the WA clade were obtained from a patient in Liberia [16] and a US soldier returning from Ghana [7]; while the CB clade included two isolates from DRC (formerly Zaire) [3,5] and one from the Republic of the Congo [6].

In the fields of ecology and biogeography, ecological niche modeling (ENM) is commonly used in studies regarding species distributions and to reconstruct their recent evolutionary and biogeographic history [17,18,19,20,21]. Several studies have shown the value of incorporating these methods in the study of the ecology and distribution of various infectious diseases, including MPX [12,22,23,24,25,26]. Ellis *et al.* [25] successfully produced suitability maps for MPX transmission using ENM; their results suggest the existence of a break in the distribution of suitable environmental conditions for MPX transmission at the Cameroon Highlands. A partition of MPX geographic range at this land feature produces two groups of MPXV that coincide with the WA and CB clades from Likos *et al.* [13].

Although the phylogenetic analysis of Likos *et al.* [13] and the ENM from Ellis *et al.* [25] consistently support the division of MPX into WA and CB clades, the small number of isolates (five) used in the former work does not provide enough resolution to determine whether the Cameroon Highlands is associated with the differentiation of these clades. Particularly, the great geographic distance separating the WA isolates (Liberia and Ghana) and the CB isolates (Republic of the Congo and DRC) makes it difficult to propose the Cameroon Highlands as the only geographic feature involved in the differentiation of MPXV clades because other potential geographic elements are also found between these isolates (e.g., Dahomey Gap, major rivers, *etc.*).

Climate oscillations during the Quaternary have caused the expansion and contraction of the geographic ranges of biomes (e.g., rainforest, savanna, tundra, *etc.*), and organisms associated with them, around the world [27,28,29]. In Africa, the rainforest contracted its geographic distribution during glacial maxima and expanded during interglacial periods, potentially impacting the distribution of MPXV and its reservoirs given the tight association between MPXV transmission events and the rainforest in Central and Western Africa [30]. The fragmentation of African rainforest through these climatic cycles could have had a role in the genetic differentiation of MPXV over several thousand years.

Here, we analyze a larger number of genomic sequences from MPXV isolates covering the known geographic distribution of MPXV to add resolution to the phylogenetic analysis and establish relationships among isolates within and between clades. Additionally, we explore the effect that climatic oscillations since the last interglacial period (*ca.* 135,000 years ago) have had on the distribution of environments suitable for transmission of MPXV and reconstruct its recent history. Finally, we integrate results from ENM with the phylogenetic evidence to identify biogeographic elements and/or geologic events that have potentially influenced the genetic differentiation of MPXV.

## 2. Materials and Methods

### 2.1. Genetic Data and Analysis

**Figure 1:**
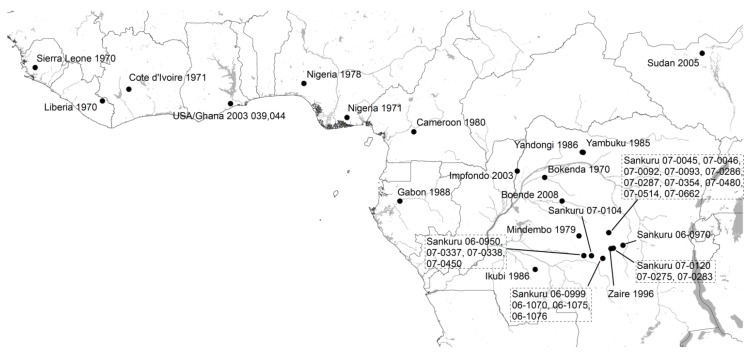
Isolates included in the phylogenetic analysis (black circles). Isolates from Kugelman *et al.* [31] are mapped to the centroid of the Health Zone where they were reported. Isolates from Copenhagen, Walter Reed, Paris, and Rotterdam are not mapped because their African origin is unknown. Isolate from Sudan is shown at the outbreak locality, but is thought to have been imported from Northern DRC.

Table 1 summarizes the MPXV isolates used in our phylogenetic analysis and provides references for the original description of the cases; they include (a) four isolates that correspond to MPXV obtained from outbreaks recorded in laboratories; (b) the five isolates included in the study by Likos *et al.*; (c) twelve isolates available from human case reports in Sub-Saharan Africa between 1970 and 2010; (d) one isolate obtained from a squirrel from Yambuku, DRC, which is the only MPXV isolate derived directly from wildlife included in this study; and (e) 23 isolates from human cases reported in Sankuru District, DRC between 2006 and 2007 [31]. In all, the 45 analyzed isolates cover the known range for MPX in Central and West Africa (Figure 1). Isolates form Nigeria, Cameroon, and Gabon are of particular interest for the present analysis since they are closer to one of the proposed biogeographic barriers (Cameroon Highlands) than the rest of the isolates from Western Africa or Congo Basin.

Genomes were sequenced using Sanger sequencing or Illumina^®^ (Illumina Inc., San Diego, CA USA) paired end sequencing. Whole genomes of MPXV isolates were aligned using MAFFT v7.017 [32]. Cowpox virus (Grisham 1990, X94355) and horsepox virus (Mongolia 1976, DQ792504) isolates were included as outgroup taxa. The original alignment was 241,258 bp in length. The first 25 kb and last 26 kb are highly variable between CPXV and other Orthopoxviruses since they contain a large number of indels; thus, they were trimmed. Subsequently, all columns containing indels were removed, resulting in an alignment of 173,804 bases from the central conserved region of the genome. A majority-rules consensus tree was estimated from the alignment of all genomes using MrBayes v3.2.2 [33,34]. Settings included a general time reversible model (lset nst = 6) with estimated stationary state frequencies and substitution rates, and a model of gamma-shaped rate variation across sites (rates=gamma). The tree search was carried out over five million generations.

A patristic distances matrix was obtained from the consensus tree and separated into the groups representing the two recognized MPXV clades. We tested the distance values within each group for normality via the Shapiro-Wilk test, compared their variances via the F-test and compared the two groups using a Student’s t-test; all statistical test were performed in R 3.1.1 [35]. MEGA v6.06 [36] was used to calculate within group uncorrelated p-distances for a subset of samples from the Lomela Health Zone in DRC.

### 2.2. Ecological Niche Models

*Environmental data*: we used a set of seven diverse and relatively uncorrelated variables [37] from the bioclimatic dataset of WorldClim [38] used in a previous monkeypox ENM study [39]: annual mean temperature, mean diurnal range, maximum temperature of the warmest month, minimum temperature of coldest month, annual precipitation, precipitation of the wettest month, and precipitation of the driest month. These variables were obtained from the WorldClim website (http://www.worldclim.org/) in their digital format at a spatial resolution of ~4 km (2.5 min) for the Last Glacial Maximum (LGM, *ca.* 21,000 years ago) and Mid-Holocene (MidHol, *ca.* 6000 years ago) periods; and ~1 km spatial resolution (30 second) for the Last Interglacial period (LIG, *ca.* 135,000 years ago) [38,40]. A buffer of 5 degrees (~555 km) of radius was created from all monkeypox case localities (see below) and was used to delineate the area in which ENMs is calibrated.

*Human case data*: we used reports of human MPX from the World Health Organization (WHO) surveillance efforts in DRC between 1970 and 1986. Case localities were georeferenced matching the patient’s village of residence to digital versions of 1:250,000 Joint Operational Graphic (JOG) topographic maps (GNS; http://earth-info.nga.mil/gns.html/) following the georeferencing procedures from MaNIS [41]; details of these procedures are included in a separate publication [39]. The aim of the present study is to identify the environmental conditions required for the disease to be transmitted from its wildlife reservoir to humans; therefore, cases suspected to have resulted from secondary transmission (*i.e.*, the disease acquired by contact with a sick person) were not included in the analysis. In all, 90 unique localities representing the entire geographic distribution of human MPX occurrence were used (white circles in Figure 3A,B). Given that most of the localities were obtained from the active surveillance efforts at the end of the smallpox eradication, this data is biased with better representation of localities in DRC. To account for this bias, we produced 25 subsets of the original dataset by reducing the density of the reported localities in DRC; specifically, we selected subsets of the original dataset in which localities were separated by a distance of 30 min (Approx. 50 km); this process produced datasets containing 41–43 localities.

*Ecological niche algorithms:* two of the most widely used ecological niche modeling algorithms (GARP and Maxent) have also been used for the study of disease transmission [22,23,26,42,43,44,45,46,47]. Both algorithms search for non-random associations between environmental conditions and the localities where the disease has been recorded; however, models may differ from one algorithm to another in their ability to estimate distributions [48] and to transfer such models into other environmental conditions or geographic areas [49], therefore, independent models were generated with each algorithm.

GARP v1.1.6 produces a model consisting of a set of rules that describe associations between environmental conditions and disease transmission events [50,51]. These models are built via an iterative process of creation, evaluation, modification, and inclusion/exclusion of rules of four basic forms (bioclimatic, atomic, negated, and logistic regression rules). This process stops when the optimization parameter changes by less than 1% from one iteration to the next, or when the maximum number of iterations is reached (1000). For each of the 25 subsets of localities, we ran 100 replicates using 50% of occurrence points to train models on environmental conditions in the present and selected the replicates with the highest performance via the “best subset” consensus approach: retaining the 20% with the lowest omission error and, from this subset, the central 50% in terms of commission error [52]. The models selected by this process (*n* = 10) were summed and evaluated using the modified ROC (E = 10%, 1000 bootstrap iterations and 50% of points), in which models performing better than random have AUC ratio values higher than 1 [53]. For a particular subset of localities, we selected the areas containing 90% of the training localities to represent the distribution of suitable environments for MPX transmission and created a binary map where 1 = suitable and 0 = unsuitable. Finally, the areas selected from each subset of localities were combined to find areas of agreement by summing the binary maps into one map with values ranging from 0 to 25.

The Maxent algorithm is based on the idea that the best solution for an unknown phenomenon maximizes entropy; thus, it calculates a probability distribution that is closest to a uniform distribution, constrained by the parameters calculated for the locality cases and a regularization parameter (β) that prevents the estimated mean values of the distribution from deviating from the observed mean value [54,55]. We used default settings in Maxent v3.2.1 (*i.e.*, regularization multiplier = 1.0, 1500 maximum iterations, 10,000 background points, convergence limit = 10^−5^) to create models for each locality subset. We evaluated the models using the modified ROC as described above and selected the areas containing all localities used for training the model for each of the 25 subsets of localities to create a binary map; these maps were combined to find areas of agreement as described above.

ENMs from both algorithms were projected into the environmental conditions during the Mid-Holocene, Last Interglacial and Last Glacial Maximum periods using the environmental datasets described in the previous section. Areas with suitable environments were selected using the methodology described above for each algorithm. The final maps for each period are the combination of the models produced by all 25 locality subsets.

## 3. Results

### 3.1. Phylogenetic Analysis

Results of the phylogenetic analysis are shown in Figure 1. The average standard deviation of split frequencies was 0.001,718. Branch lengths between MPXV isolates are shown to scale. The Bayesian analysis grouped 11 isolates in the WA clade and 34 in the CB clade. Geographically, the WA clade isolates correspond to those cases reported from Nigeria, Ghana, Liberia, Cote d’Ivoire, and Sierra Leone, including the four isolates from outbreaks in captive animals; while the CB clade includes all isolates from DRC, Republic of the Congo, Cameroon, and Gabon. Although four polytomies resulted from the phylogenetic analysis, node support was very high (0.99–1.0) throughout most of the tree except for the polytomy that includes groups I-IV, which has support of 0.868. Eight isolates from Lomela Health Zone are included in a polytomy within group III (JX878413, JX878414, JX878415, JX878416, JX878421, JX878422, JX878427, and JX878428), with an average distance between them of 0.002%. Comparison of within clade patristic distances showed that branches in the CB clade are significantly shorter than branches within the WA clade (Shapiro-Wilk *p* < 0.001 in both clades, F-test *p* = 0.001, and *t*-test [unequal variance] *p* < 0.001). The two Nigerian isolates group together in spite of having high genetic differentiation between them and are from a group that is sister to the one formed by the remaining isolates of the clade; in addition, all isolates obtained from captive animals form a single monophyletic group.

**Figure 2:**
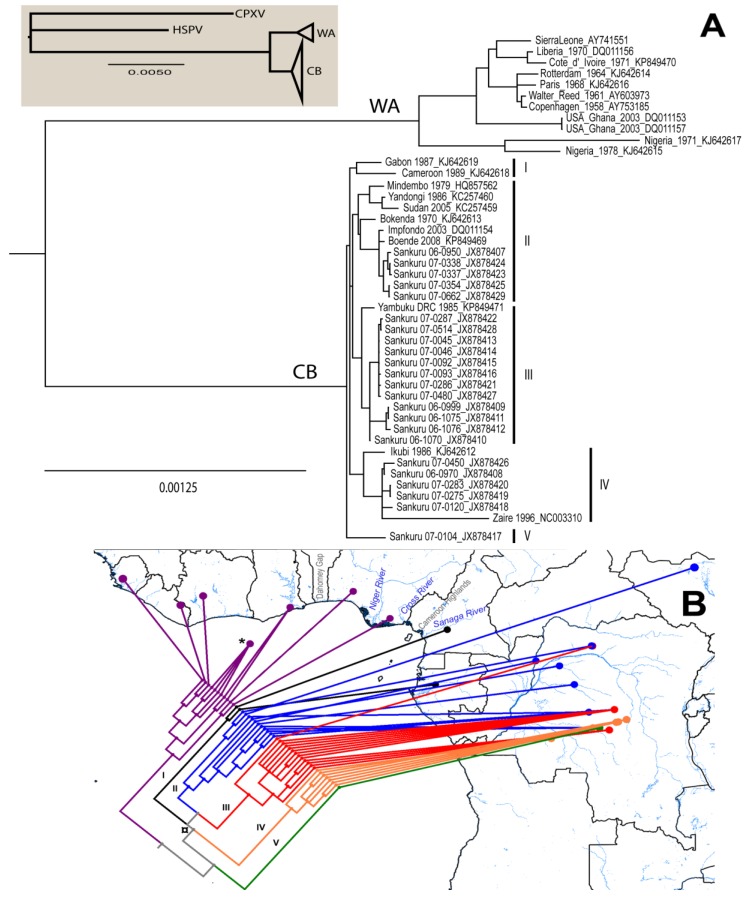
(**A**) Majority-rules consensus tree. Clade credibility values for all nodes are 0.99–1.0, except from one polytomy indicated in the text (**¤**). Branch lengths are shown to scale. CB = Congo Basin Monkeypox clade; WA = West African Monkeypox clade. Groups are indicated by the roman number in parenthesis; (**B**) Map showing major geographic features of the area and the distribution of isolates used in the phylogenetic analysis, colors indicate groups: I = black, II = blue, III = red, IV = orange, V = green, and WA = purple. Isolates from Copenhagen, Walter Reed, Paris and Rotterdam are mapped to a point in the sea indicated with an asterisk (*) because their African origin is unknown.

Within the CB clade, five monophyletic groups were formed after the phylogenetic analysis: group I includes the two western most isolates from this clade: Cameroon 1989 and Gabon 1987; group II contains 11 isolates, five from Sankuru District (JX878407, JX878423-25, and JX878429), one from South Sudan (Sudan 2005), one from the Republic of the Congo (Impfondo 2003), and four from other parts of DRC (Boende 2008, Bokenda 1970, Yandongi 1986, and Mindembo 1979); group III is the closest sister group to group II, twelve of the thirteen isolates in this group are from Sankuru District (JX878409-16, JX878421-22, JX878427-28), the remaining one is from Yambuku; six of the seven isolates in group IV are from Sankuru District (JX878408, JX878426, JX878417-20, and Sankuru 1996), the remaining one is Ikubi; group V only has one isolate from Sankuru District (JX878417). The groups are indicated in Figure 2 and Table 1.

**Table 1:** Isolates used in the phylogenetic analysis, locality of report, publication source and accession number. ** = Used by Likos *et al.* [13]. ++ = Used in Kugelman *et al.* [31]. Roman numerals in parenthesis after the isolate name indicate the Congo Basin group to which they belong based on the phylogenetic analysis.

Isolate Name	Location	Source	Accession #
Ikubi 1986 (IV)	Ikubi, DRC (Zaire)	[4]	KJ642612
**++ Zaire 1996 (IV)	Akungula, DRC (Zaire)	[5]	NC_003310
Yambuku DRC 1985 (III)	Yambuku, DRC (Zaire)	[56]	KP849471
**++ Mindembo 1979 (II)	Mindembo, DRC (Zaire)	[3]	HQ857562
Yandongi 1986 (II)	Yandongi, DRC	[4]	KC257460
Bokenda 1970 (II)	Bokenda, DRC (Zaire)	[1]	KJ642613
Boende 2008 (II)	Boende, DRC	Unpublished	KP849469
**++ Impfondo 2003 (II)	Impfondo, ROC	[6]	DQ011154
Sudan 2005 (II)	Nuria, South Sudan	[57]	KC257459
Cameroon 1989 (I)	Ekoumdouma, Cameroon	[58]	KJ642618
Gabon 1987 (I)	Gabon	[59]	KJ642619
Nigeria 1971	Ihie, Nigeria	[16]	KJ642617
Nigeria 1978	Omifunfun, Nigeria	[3]	KJ642615
++ USA/Ghana 2003_039	Ghana	[7]	DQ011157
**++ USA/Ghana 2003_044	Ghana	[7]	DQ011153
++ Copenhagen 1958	Copenhagen	[60]	AY753185
++ Walter Reed 1961	Walter Reed	[61]	AY603973
Paris 1968	Paris	[62]	KJ642616
Rotterdam 1965	Rotterdam	[63]	KJ642614
Cote d’Ivoire 1971	Cote d’Ivoire	[64]	KP849470
**++ Liberia 1970	Liberia	[16]	DQ011156
++ Sierra Leone 1970	Sierra Leone	[16]	AY741551
++ Sankuru 06-0950 (II)	Kole Health Zone, DRC	[31]	JX878407
++ Sankuru 07-0337 (II)	Kole Health Zone, DRC	[31]	JX878423
++ Sankuru 07-0338 (II)	Kole Health Zone, DRC	[31]	JX878424
++ Sankuru 07-0450 (IV)	Kole Health Zone, DRC	[31]	JX878426
++ Sankuru 06-0999 (III)	Vangakete Health Zone, DRC	[31]	JX878409
++ Sankuru 06-1075 (III)	Vangakete Health Zone, DRC	[31]	JX878411
++ Sankuru 06-1076 (III)	Vangakete Health Zone, DRC	[31]	JX878412
++ Sankuru 06-1070 (III)	Vangakete Health Zone, DRC	[31]	JX878410
++ Sankuru 07-0045 (III)	Lomela Health Zone, DRC	[31]	JX878413
++ Sankuru 07-0046 (III)	Lomela Health Zone, DRC	[31]	JX878414
++ Sankuru 07-0092 (III)	Lomela Health Zone, DRC	[31]	JX878415
++ Sankuru 07-0093 (III)	Lomela Health Zone, DRC	[31]	JX878416
++ Sankuru 07-0286 (III)	Lomela Health Zone, DRC	[31]	JX878421
++ Sankuru 07-0480 (III)	Lomela Health Zone, DRC	[31]	JX878427
++ Sankuru 07-0514 (III)	Lomela Health Zone, DRC	[31]	JX878428
++ Sankuru 07-0287 (III)	Lomela Health Zone, DRC	[31]	JX878422
++ Sankuru 07-0354 (II)	Lomela Health Zone, DRC	[31]	JX878425
++ Sankuru 07-0662 (II)	Lomela Health Zone, DRC	[31]	JX878429
++ Sankuru 06-0970 (IV)	Katako Kombe Health Zone, DRC	[31]	JX878408
++Sankuru 07-0120 (IV)	Djalo-Ndjeka Health Zone, DRC	[31]	JX878418
++Sankuru 07-0275 (IV)	Djalo-Ndjeka Health Zone, DRC	[31]	JX878419
++Sankuru 07-0283 (IV)	Djalo-Ndjeka Health Zone, DRC	[31]	JX878420
++Sankuru 07-0104 (V)	Bena-Dibele Health Zone, DRC	[31]	JX878417

### 3.2. Ecological Niche Model

**Figure 3:**
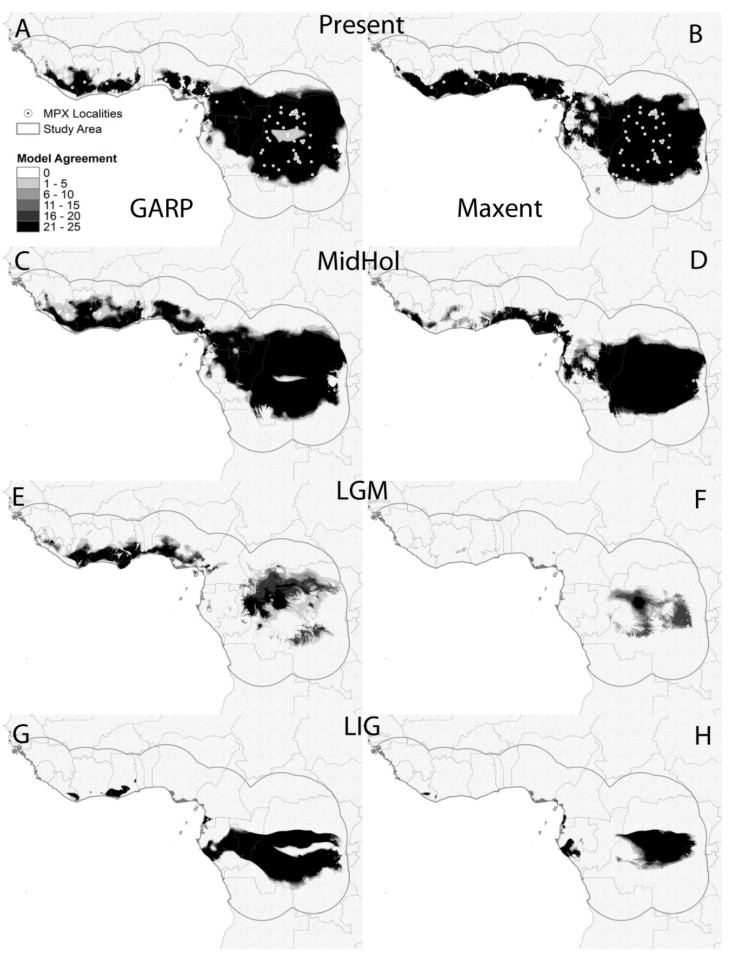
Model agreement maps from GARP (**left column**) and Maxent (**right column**) based on the 25 locality subsets; darker shade = higher agreement. Models projected onto present day environmental conditions (**A**,**B**), Mid-Holocene (MidHol: **C**,**D**), Last Glacial Maximum (LGM: **E**,**F**), and the Last Interglacial period (LIG: **G** and **H**). White circles represent MPX localities used for model development.

ENM projections into present day environmental conditions are similar to models presented in previous works [25,39] and identify areas in Central and Western Africa in which MPX is known to occur. Models based on each of the 25 locality subsets performed better than random expectations with modified ROC AUC values between 1.31 and 1.42 for GARP (average = 1.37, standard deviation = 0.03); and between 1.33 and 1.48 for Maxent (average = 1.42, standard deviation 0.04). These models are able to capture those environmental conditions that are suitable for MPX transmission with high confidence. ENM maps presented some differences: (a) GARP models predicted a larger area of suitable environmental conditions in Cameroon and Gabon than the models produced by Maxent; (b) Maxent models show a more uniform distribution of suitable conditions in West Africa, while GARP shows fragmentation; and (c) GARP models show an area of low model agreement in the middle of the Congo River Basin (Figure 3A,B).

GARP model projections onto climatic conditions during the Mid-Holocene show more connection between areas with suitable environments for MPXV in West Africa and also between those suitable areas in Nigeria and Central Africa; the most evident discontinuity of suitable areas separates such areas in costal Nigeria from the rest of West Africa and is located in Benin (Figure 3C). Maxent, however, shows a noticeable reduction of suitable areas in West Africa concentrated in the coasts of Nigeria, Togo and Benin with some smaller suitable areas in Sierra Leone, Liberia and Ghana (Figure 3D). ENM projections onto environmental conditions during the LGM with both algorithms identified smaller areas with suitable environments for MPX transmission. The area with highest model agreement for Maxent is located in western DRC with a few other areas with lower model agreement within this country; however, only few small areas in West Africa were identified as suitable by a few models (Figure 3F). Areas of high model agreement for GARP are also restricted to small areas in northern and western DRC, as well as along the coast throughout West Africa (Figure 3E).

Finally, ENM projections onto environmental conditions during the LIG identified a larger and more continuous area of model agreement for both algorithms than that for the LGM located in central Africa with a few small patches along the coast in West Africa for GARP and Gabon for Maxent (Figure 3G,H).

## 4. Discussion

Our phylogenetic analysis separates the MPXV isolates into two major groups that correspond to the previously identified WA and CB clades. The eastern most isolate of the WA clade (Ihie, Nigeria) is between the Niger River (west) and the Cross River (east); while the western most isolate of the CB clade (Ekondouma, Cameroon) is south of the Sanaga River (Figure 2B). These rivers (Cross and Sanaga) have been identified as biogeographic barriers for mammal species such as chimpanzees of the genus *Pan* [65], flying squirrels [66], and mice of the genus *Praomys* [67]. The two isolates from Nigeria are on opposite sides of the Niger River and they are more genetically divergent than other pairs of isolates from the West African clade, adding support to the idea that rivers could play a role in the differentiation of MPXV.

The Cameroon Highlands are also located between the Cross and Sanaga Rivers; they are recognized as a high biodiversity ecoregion where the dominant vegetation types are tropical and subtropical moist broadleaf forest [68], representing a change in altitude and dominant vegetation coverage from the lowland evergreen forest of the Congo Basin. Although the presence of these geographic features suggest their involvement on the genetic divergence of the two MPXV clades; with the data available to this point, it is not possible to determine which features are currently acting or previously acted as dispersal barriers for MPXV or its reservoir species.

The Dahomey Gap is a savanna corridor that interrupts the West African rain forest in Togo, Benin, and Eastern Ghana [69], which has been hypothesized to act as a barrier to dispersal of mammals [70,71]. Given that most human MPX cases have been associated with the rainforest [3,72,73], we would expect the Dahomey Gap to be a dispersal barrier for MPXV because the dominant land cover and climatic conditions would not be suitable for the reservoir or transmission of the virus based on ENMs. Results of phylogenetic analyses support this by revealing separate groups for isolates located west (Ghana, Cote d’Ivoire, Liberia, and Sierra Leone) and east of the Dahomey Gap (Nigeria). Palynological analyses indicate that an abrupt climatic change into drier conditions favored the establishment of the savanna in this area starting around 4500 years ago [69].

Consistent with the Pleistocene refuge theory [74,75], ecological niche models predicted smaller areas with suitable conditions for MPXV transmission during the LGM (21,000 years ago), especially in the Congo Basin, representing a potential bottleneck for MPXV in the Congo basin. The posterior expansion of the rainforest driven by warmer and more humid conditions in the area, may have allowed MPXV and/or its reservoir(s) to also expand its geographic range, potentially leading to a rapid diversification of the virus, as shown by the relatively short branches of the CB clade and the polytomy that includes groups I to IV in the phylogenetic tree (Figure 2A). As the niche model of MPXV in Western Africa during LGM was less restrictive, it would be expected to maintain a larger portion of its genetic variation.

In the present study, we first identified potential biogeographic barriers for MPXV that could be related to the CB-WA split. Further studies are necessary to determine whether the presence of a river, change in elevation, or change in the dominant vegetation cover is involved in the genetic differentiation of MPXV. The addition of MPXV isolates from the area between the Sanaga and Cross rivers would be ideal; however, cases of human or wildlife MPX have not been reported from this area. Second, we propose that the CB clade is a group with very recent diversification, possibly explained by the colonization of a bigger area with suitable conditions (refuge theory); however, dating the times of differentiation between and within clades is not possible with our current dataset. Tying MPXV cladogenesis to geologic or climatic events is a subject of future efforts. Additional field studies that result in the isolation of MPXV or the finding of serological evidence of infection with this virus in wildlife could be key to better understanding its natural history and biogeography.
